# The role of intestinal stem cell within gut homeostasis: Focusing on its interplay with gut microbiota and the regulating pathways

**DOI:** 10.7150/ijbs.72600

**Published:** 2022-08-08

**Authors:** Haoming Luo, Mingxing Li, Fang Wang, Yifei Yang, Qin Wang, Yueshui Zhao, Fukuan Du, Yu Chen, Jing Shen, Qianyun Zhao, Jiuping Zeng, Shengpeng Wang, Meijuan Chen, Xiaobing Li, Wanping Li, Yuhong Sun, Li Gu, Qinglian Wen, Zhangang Xiao, Xu Wu

**Affiliations:** 1 Laboratory of Molecular Pharmacology, Department of Pharmacology, School of Pharmacy, Southwest Medical University, Luzhou 646000, Sichuan, China.; 2 Cell Therapy & Cell Drugs of Luzhou Key Laboratory, Luzhou 646000, Sichuan, China.; 3 South Sichuan Institute of Translational Medicine, Luzhou 646000, Sichuan, China.; 4 State Key Laboratory of Quality Research in Chinese Medicine, University of Macau, Macao, China.; 5 Department of Oncology, Affiliated Hospital of Southwest Medical University, Luzhou 646000, Sichuan, China.

**Keywords:** Intestinal stem cell, Intestinal barrier, Gut microbiota, Intestinal homeostasis, Signaling pathway

## Abstract

Intestinal stem cells (ISCs) play an important role in maintaining intestinal homeostasis via promoting a healthy gut barrier. Within the stem cell niche, gut microbiota linking the crosstalk of dietary influence and host response has been identified as a key regulator of ISCs. Emerging insights from recent research reveal that ISC and gut microbiota interplay regulates epithelial self-renewal. This article reviews the recent knowledge on the key role of ISC in their local environment (stem cell niche) associating with gut microbiota and their metabolites as well as the signaling pathways. The current progress of intestinal organoid culture is further summarized. Subsequently, the key challenges and future directions are discussed.

## 1. Introduction

As a natural defense system, the intestinal luminal surface is covered by just one layer of epithelium. However, that layer is an effective barrier that guards against pathogens and toxins that may be harmful to the host 1-3. There are several mechanisms that may participate in the disruption of barriers in the gastrointestinal (GI) tract, including inflammation, infection, *etc.*, and lead to dysregulation of intestinal homeostasis. Intestinal homeostasis is maintained by a combination of the GI luminal epithelium, gut microbiota, and intestinal stem cells (ISCs). Recent research has focused on the mechanisms of controlling intestinal homeostasis both in health, injury and recovery conditions. Fortunately, recent technological advancements such as intestinal organoids and organ cultures of the gut have allowed us to gain a deeper understanding of this system.

ISCs in the crypts of the intestines are rare, non-quiescent, undifferentiated cells, which play a crucial role in regulating the homeostasis and regeneration of tissues. Each crypt contains four to six pluripotent stem cells 4, 5. Nevertheless, a key question is how to coordinate the mechanisms of ISC renewal and differentiation. The microbiota found in the GI tract is made up of various microbial communities which interact with the host at the intestinal ecotone. Gut microbiota influences the intestinal epithelial development by interfering with ISCs through the abundance of commensal bacteria and invasive organisms, as well as through metabolites produced by microorganisms. A balance is assumed to be achieved among ISCs, gut microbiota and other factors within the gut homeostasis.

The aim of this review is thus to review the recent knowledge on the key role of ISC in their local environment (stem cell niche) associating with gut microbiota and their metabolites as well as the signaling pathways. The current development of intestinal organoid culture is further summarized. The key challenges and future directions are further discussed.

## 2. ISCs in maintaining intestinal barrier

### 2.1 Intestinal structure and intestinal homeostasis

The GI tract, which has a surface area of approximately 250 cm^2^, is not only the primary site of nutrient absorption and processing, but also the body's largest endocrine organ 1. Mucosal surfaces of the GI tract are surrounded by epithelial cells (ECs), creating a natural barrier of intercellular connections that separates the internal and external environments. This barrier selectively absorbs nutrients and electrolytes while simultaneously prevents the passage of potentially harmful substances (antigens, toxins, and microbial by-products) into the body. For the epithelial barrier to remain intact, many defense systems are involved, that the immune system develops normally, and that tolerance to dietary antigens and intestinal microbiota is maintained. This allows the GI tract to function as a robust barrier against potential hazardous infections and toxins 2, 3.

**Figure 1:**
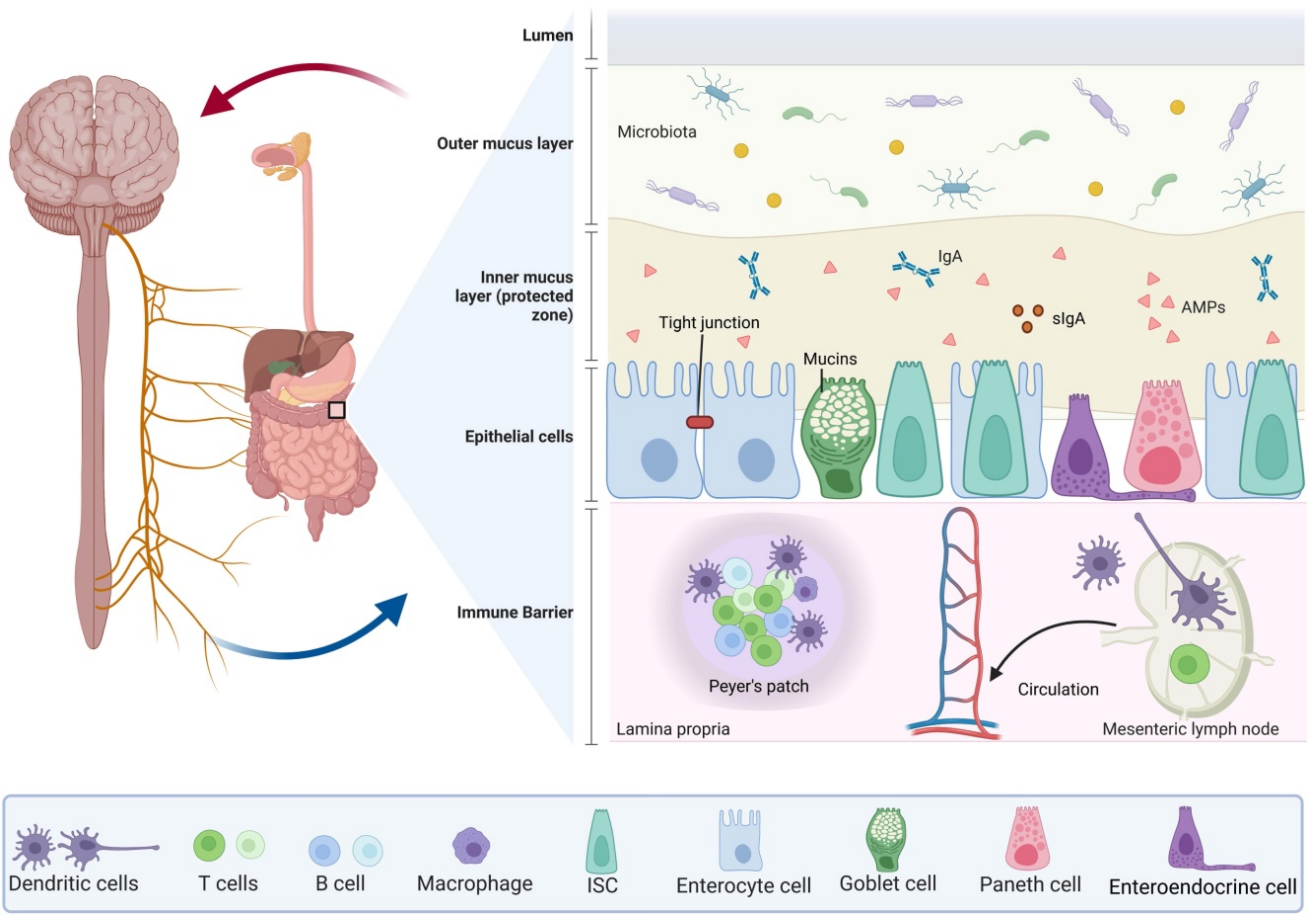
** The components in intestinal structure for maintaining intestinal barrier.** The intestinal barrier consists of two layers: the outer layer consists of the intestinal microbiota (including bacteria, viruses, and pathogens), the mucus layer and the ECs, working together to form a physical barrier against infection. The mucus layer contains sIgA produced by Paneth cells and mucins produced by goblet cells, in addition to intercellular TJs. The inner layer provides a chemical barrier to prevent the spread of bacteria into the host tissue. The GALT is an important immune system in the gut and is composed of the Peyer's patches, the interdigitating lymphocytes, plasma cells and lymphocytes presented in the lamina propria, and mesenteric lymph nodes. All of these are closely linked to the blood circulation in the body.

The intestinal barrier is composed of two layers (Figure 1): the outer layer serves as a physical barrier, preventing bacteria from adhering and regulating decidual cell spread into the host tissue; and the internal layer provides a chemical barrier, preventing the spread of bacteria to the host tissue. The intestinal microbiota, the mucus layer, and ECs work together to form the physical barrier against infection. For example, there is competition between intestinal flora and vector pathogens for resources in the GI tract. These colonies of bacteria can process molecules that are essential for mucosal integrity, and regulating immune activity in the deep barrier, among other things. In addition to assisting in the retention of antimicrobial peptide-rich mucus, the mucus layer also helps to prevent mucosal adhesion and subsequent microbial invasion across the epithelium 6, 7. Furthermore, the mucus layer contains secretory immunoglobulin A (sIgA) produced by a type of plasma cell as well as antimicrobial products produced by the Paneth cells which include phospholipids, negatively charged mucins, and peptides such as trilobal factor family (TFF) peptides and antimicrobial peptides that are active against bacteria, yeast, fungi, viruses, and tumor cells. IgA and other antimicrobial products are essential to maintain gut homeostasis as they are necessary for preventing gut inflammation to occur 8. In addition, the intestine contains tight junctions (TJs), which are composed of a network of transmembrane protein chains that include intact membrane proteins (claudin family proteins, which are involved in selective ion permeation as the primary mode of transmembrane epithelial transport, and another transmembrane proteins called occludins), binding complex proteins, as well as intracellular signaling molecules 3, 9. The TJs are controlled and regulated by extracellular signaling molecules. Upon the occurrence of pathogens and commensal microorganisms within the inner layer, TJs act as an immune barrier, organizing immune tolerance and pathogen response 10. In addition, the gut-associated lymphoid tissue (GALT) is a key immunological system in the gut which is comprised of Peyer's patches, interdigitating lymphocytes, plasma cells and lymphocytes present in the lamina propria, and mesenteric lymph nodes. GALT is divided into two types: organized and diffuse GALT. Organized GALT initiates the immune response to the massive antigen exposure, whereas diffuse GALT is the effector. Diffuse GALT is made up of numerous leukocyte populations that are dispersed on either side of the basement membrane 11.

It is common for intestinal homeostasis to be dysregulated when barrier integrity is disrupted. Maintaining gut homeostasis is achieved through a delicate interplay among ECs, gut microbiome, immune system, as well as ISCs 11-13. Several findings have shown that ISCs are regulated and influenced by the microenvironment (stem cell niche) derived from specialized epithelial and mesenchymal cells. ISCs are involved in tissue homeostasis and regeneration after injury or inflammation 4, 13, 14. As expected, intestinal ECs, commensal bacteria, immune system and ISCs work together to maintain the luminal environment in the intestine. Notably, disruption of intestinal homeostasis can result in GI inflammation, such as inflammatory bowel disease (IBD) 8. The epithelial barrier breach associated with IBD is characterized by an increase in EC death, a decrease in TJ protein expression, and a deficiency in immune response 15, which also contributes to initiation of diseases of other systems.

### 2.2 The role in ISCs in maintaining gut barrier

ISCs in the crypts are undifferentiated and are involved in tissue homeostasis and regeneration after injury or inflammation 4, 13, 14. ISCs near the crypt base is the source of all putative mitotically developed cells. Back in 2009, Walker *et al.* pointed out that stem cells that maintain tissue homeostasis were regulated and supported by the surrounding microenvironment, known as the stem cell niche 16. In 2017, Meran *et al.* formally introduced the concept of the ISC niche, which includes nearby Paneth cells, neuronal cells, smooth muscle cells and stromal cells, as well as the extracellular matrix together 17. Moreover, the intestinal epithelium is a site of rapid cell renewal and is highly in need of regulation by the ISCs located at the base of the crypts. The maintenance of ISC activity is influenced by different paracrine ecotropic factors and a number of cellular signals such as Wnt, R-spondin, Notch and bone morphogenetic protein (BMP), as well as by inflammation, gut microbiota and diet. Indeed, following injury, the ISC niche stimulates EC regeneration 18. When Noggin (a BMP inhibitor) and downstream Wnt signaling targets (*e.g.* EPhB and C-Myc) are overexpressed in the embryonic intestinal epithelium, exceptional crypts are formed with the assistance of villi that are perpendicular to the crypt-villi axis 19, 20. For example, each intestinal crypt contains four to six long-lived pluripotent stem cells, which are actively dividing during the day, rather than being quiescent 4, 5.

ISCs replenish themselves by mitosis, dividing into equal stem mobile clones over time. Such division ensures constant stem cell diversification and differentiation. ISCs are required to sustain themselves for an extended period while also provide upward movement to all differentiated types of tissue in question; this is referred to as their "stemness". Stem cells must also be able to provide upward movement to all differentiated mobile phone types of tissue in question. According to Barker *et al.*, the direct progeny of stem cells, known as TA cells (transit-amplifying cells), would unexpectedly dilute any integrated DNA markers used to demonstrate their stemness on remote adult stem cells. A common method of achieving "stemness," therefore, is the long-term storage of DNA labels. When tissue has been severely damaged, stem cells go through mitosis to maintain DNA markers in the tissue. On the contrary, because they are unable to proliferate, DNA labels may continue to be retained by terminally differentiated cells longer than they would in stem cells 4, 14.

**Figure 2:**
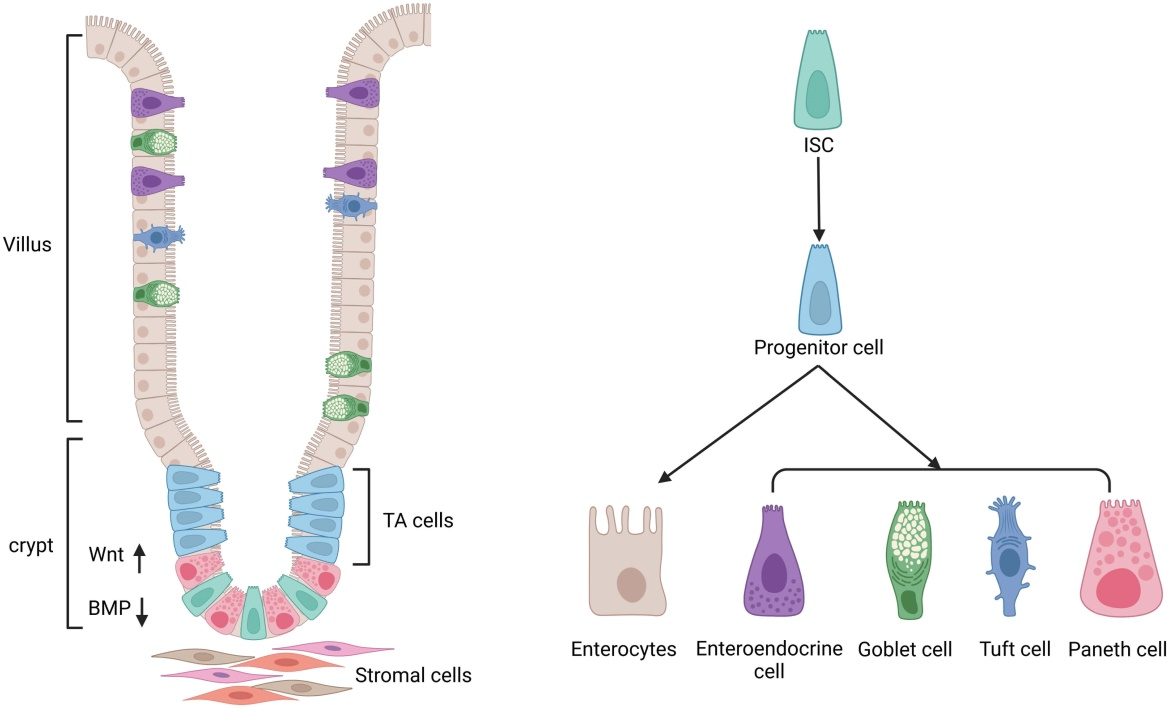
** ISC niche and its differentiation.** The regulation of the activity of ISCs is dependent on the stem cell niche, including surrounding stromal cells and signaling molecules. The intestinal epithelium is made up of one type of absorptive cell and four types of secretory cells. Transit-amplifying (TA) cells act as direct descendants of ISCs, including secretory and absorptive progenitors, which can give rise to stem-like cells following stem cell injury. Secretory progenitors are differentiated into Paneth cells, goblet cells, tuft cells, and enteroendocrine cells, while absorptive progenitors are differentiated into enterocytes.

The small intestinal epithelium is made up of one type of absorptive cell and four types of secretory cells (Goblet cells, enteroendocrine cells, tuft cells, and Paneth cells), which are differentiated from stem cells and generated from the endoderm 4, 13, 14, 21 (Figure 2). Most ECs are goblet cells, which account for 10-15 percent of small intestine ECs and 50 percent of colonic ECs. Goblet cells are involved in the production and secretion of mucus, creating a protective barrier for ECs. A recent study showed that ST6GALNAC1 (ST6), a saline acid transferase specifically expressed in goblet cells is essential for mucus integrity and protecting from microbial degradation 22. It is estimated that enteroendocrine cells account for 1% of the epithelium of the intestine. These cells are dispersed among the mucus as character cells, which are responsible for the production and release of hormones. Tuft cells are a type of secretory cell that has only recently been discovered in the intestines of newborn mice and are thought to be responsible for their secretion 23. Only tuft cells categorize cyclooxygenase and express the style protein (α-gustducin, Trpm5) in response to vitamins in the intestinal lumen 23. They are also the only ECs that secrete opioids in response to vitamins 23. In comparison with other ECs, which appear later and coincide with the appearance of the crypt 13, Paneth cells synthesize and exude antimicrobial peptides into the intestinal lumen. To preserve their capacity to proliferate and self-renew, ISCs rely on the niche cells surround them (Figure 2). When ISCs continually self-renew, they produce progenitor-transporting amplifying cells, and progenitor cells undergo additional mobile divisions before maturation and differentiation. Under normal circumstances, the regeneration of the ECs takes 1-2 weeks in drosophila, whereas in mice it takes only 5-7 days 24, 25.

Since ISCs allow for the continuous replacement of intestinal ECs, they are critical for maintaining the mucosal barrier, which is a mechanism that protects the intestinal barrier from pathogen invasion and bodily damage in pathological or physiological conditions that activate chronic or acute inflammation. Irritable bowel syndrome is a disorder related to the gut, involving abnormal GI motility, abnormal absorption or secretion, and visceral hypersensitivity in the presence of a stressful environment. Actually, these symptoms may also be associated with a low density of enteroendocrine cells, which is caused by a low density of ISCs 21. Potton *et al.* had previously discovered that Musashi-1 in mice intestinal tissue was an early marker of stem cells and progenitor cells, and is expressed only in damaged tissue (*e.g.* tumors) 5. A recent study demonstrated that deficiency of SETDB1, a histone methyltransferase that mediates the trimethylation of histone H3 at lysine 9, in ISCs led to irreversible disruption of epithelial barrier homeostasis, promoting intestinal inflammation and causing severe IBD 26. Additionally, Zhang *et al.* discovered that ISCs controlled the stability in the GI environment through tumor-suppressive autophagy 27.

## 3. Interplay of gut microbiota and ISCs

Keeping the intestinal lumen stable can be a complex task, which necessitates interaction among the ECs lining the lumen, ISCs, and gut microbiota. The intestinal microbiota comprises a variety of microbial communities, which include viruses, bacteria, and fungi, which is essential for regulating GI function and immune homeostasis 28, 29.

In healthy state, microbiota variability, as reflected in intestinal bacterial diversity and microbial growth rates, is mainly correlated with genetic, environmental and individual differences 30-34. Notably, intestinal bacteria imbalance contributes significantly not only to intestinal diseases such as IBD, but also to some systemic chronic conditions such as obesity, cardio-metabolic disease (CMD), type 2 diabetes (T2D), metabolic syndrome, and malnutrition 35-37. Changes in the abundance of microbiota in healthy and diseased states are shown in Table 1. Firstly, intestinal microbiota has been shown to be abnormally grown under diseases. For instance, Liu *et al.* found that the abundance of *Bacteroides thetaiotaomicron* was significantly reduced in obese patients and inversely related to serum glutamate concentrations 38. Allin *et al.* discovered a decrease in the abundance of *Clostridium spp*. and *Akkermansia muciniphila*, whereas the abundance of *Ruminococcus*, *Sutterella* and *Streptococcus* increased in the gut of T2D patients 39. Moreover, microbiota was demonstrated as one of main contributors of disease onset and progression. Altered intestinal microbiota profile led to excess endogenous alcohol production, and increased pro-inflammatory factors which contributed to non-alcoholic fatty liver disease (NAFLD) 40-42. It should be noted that dysbiosis (imbalance of intestinal microbiota) has been regarded as onset of most chronic diseases, due to unhealthy diet or environmental changes. Both gain and loss of function dysbiosis are expected to cause intestinal injury and chronic inflammation, which, coordinating with other factors or solely, contributes to disease.

**Figure 3:**
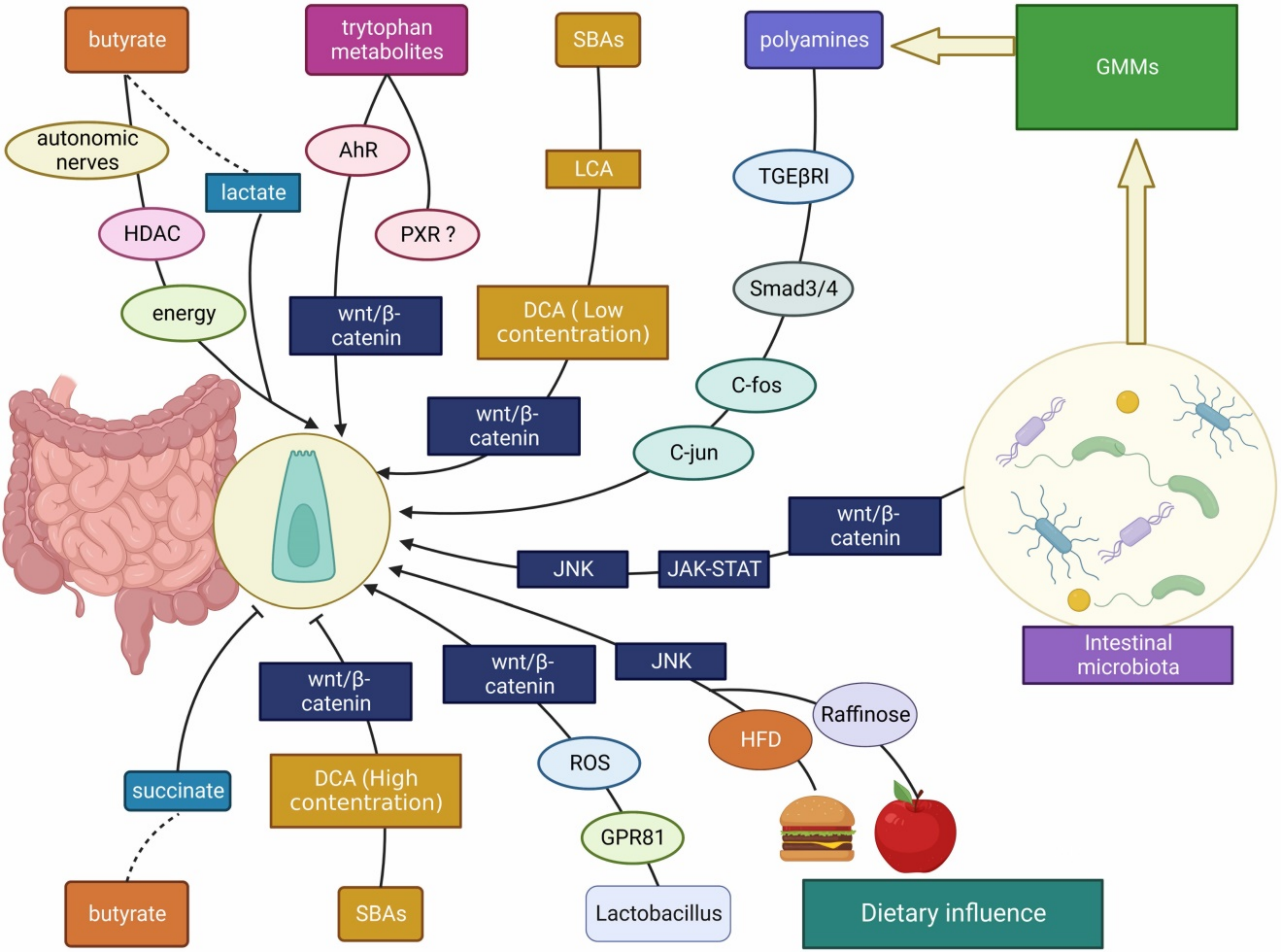
** The interplay between gut microbes/ microbial metabolites/ dietary factors and ISCs.** The intestinal microbiota, gut microbial metabolites (including SCFAs, SBAs, tryptophan metabolites, *etc.*) and dietary influences (including HFD, polysaccharides, *etc.*) affect the development of the intestinal mucosa mainly through Wnt/β-catenin, JNK, JAK-STAT and other signaling molecules to promote/ inhibit ISC proliferation.

Throughout the last decade, researchers have discovered that gut bacteria show a complex interplay with ISCs, which plays an important role in gut health. Therefore, this section aims to summarize the recent developments in regulating intestinal microbiota/ derived metabolites-ISC axis (Figure 3). The effects of gut microbes and gut microbial metabolites (GMMs) on ISCs in different gut states are displayed in Table 2.

### 3.1 Intestinal microbiota and ISCs

Many studies have established a link between the gut microbiota and ISCs, revealing clear communication at the niche level. ISCs are often used as a tool, which is due to its ability to promote a continuous renewal of the intestinal ECs. It has been shown that the intestinal microbiota has an impact on how the intestinal ECs grows and develops. To discover whether early intestinal microbiota contributes to the development of the intestinal tract, Yu and colleagues examined the differentiation and formation of TJs in four different epithelial cell lines 29. Early intestinal bacteria have been demonstrated to be a cause of villi height and crypt formation 29. Particularly, early microbes from a preterm infant with normal weight gain mediated increased villus height and crypt depth, elevated cell proliferation, high numbers of goblet cells and Paneth cells, and enhanced TJs 29.

Remarkably, intestinal microbiota exerts varied influences on ISCs during different intestinal states (healthy, injury and regenerative conditions). In a steady-state condition, normal gut microbiome, featuring with balanced beneficial and potential harmful bacteria, helps to maintain gut health. It seems that gut microbiota under healthy condition probably does not give specific advantage to ISCs. Schoenborn* et al.* demonstrated that no differences in ISC numbers or cycling activity were observed in the small intestine of germ-free and conventionally raised mice, which suggested that enteric microbiota did not impact ISCs under a normal condition 43. Another study compared the transcriptome/proteome expression phenotypes of primary organoids established from germ-free and microbiota-associated mice, indicating there were no significant differences 44.

In contrast, accumulated evidence confirms that intestinal microbiota is essential for ISC activity during injury and repair states. The cytosolic bacterial peptidoglycan sensor Nod2 was found highly expressed in ISCs linking microbes to gut epithelial regeneration 45. Nod2 knockout mice were viable and did not show any particular difference compare to wide-type mice 45. However, under stress conditions, the presence of microbiota made ISCs more prone to respond to microbiota-secreted metabolites in a Nod2 dependent manner, protecting from injury. The results indicate that gut microbiota may not influence ISCs in normal condition, but robustly exerts effect on ISCs function and EC renewal and repair.

Alteration of intestinal microbiota is characterized as either loss of function and/or gain of function. Opportunistic pathogens and their activities could be obtained or overgrow to promote diseases such as infectious diseases, which is termed gain of function dysbiosis. Loss of function dysbiosis is often due to the suppression of beneficial bacteria and their activities, which can be linked to diseases like IBD and obesity. Changes in the composition of the intestinal microbiota can cause abnormal reprogramming of ISCs via a variety of mechanisms including genetic and epigenetic failure, impaired metabolism, ISC stability, and abnormal immune activation 46. The reciprocal symbiotic connection between the host and microbiota under physiological conditions could be lost as a result of changes in the microbial composition and/or the host's genetic susceptibility to infection 46. Tao *et al.* found that the intestine's relative quantity of beneficial and invading bacteria was directly related with the production and expression of inflammatory cytokines, which interacted to regulate immune function 47. Notably, it is speculated that bacteria, through their contact with the gut epithelium, have an impact on the production and assembly of TJs and affecting host genome and immunity, thereby controlling the permeability and reprogramming of the epithelium 47. Mucin-degrading bacteria (such as *Akkermansia muciniphila*) have been shown to be critical for ISC-mediated epithelial growth and intestinal environment stability in a recent study 48. Wu *et al.* showed that *Lactobacillus reuteri* induced intestinal epithelial cell proliferation to repair epithelial damage through an increase in R-spondins and reduced intestinal pro-inflammatory cytokine secretion and serum lipopolysaccharides (LPS) concentrations, thereby activating the Wnt/β-catenin pathway 49. Furthermore, Buchon* et al.* demonstrated that both invasive bacteria and commensal bacteria could affect adult ECs renewal, demonstrating the critical role of ISCs in maintaining the GI environment's stability and host defense. However, the gut microbiota-intestinal epithelium interaction is still largely unknown due to unknown variables and processes. This is potentially due to the fact that a large number of microbes exist in the gut and are involved in complex regulations.

Given the role of gut microbiota and ISCs in gut health, it has been suggested that manipulation of gut microbiome via colonization of specific beneficial bacteria strains or dietary supplementation of prebiotics may be beneficial in certain intestinal diseases. Chen *et al.* demonstrated that *Clostridium butyricum* inhibited the growth of high-fat diet (HFD)-induced intestinal tumor development in *Apc*^min/+^ mice through modulation of Wnt signaling and alteration of intestinal microbiome 51. The researchers showed that *Bacillus subtilis* not only attenuated the inflammatory response, stimulated the proliferation of ISCs, facilitated the repair of the intestinal barrier, but also restored intestinal flora balance. Ferments of *B. subtilis* relieved IBD 52. Moreover, *Chrysanthemum morifolium* polysaccharides have been shown to be useful in the treatment of ulcerative colitis (UC) by enhancing the establishment of healthy gut bacteria, restoring balance to the intestinal micro-ecological environment, and regenerating the immune system 47. Although it is demonstrated that manipulating gut microbiota presents a promising strategy for maintaining gut health, the methods for precision regulation of gut microbiota are still lacking. The homeostasis of gut is maintained by balance of different microbes. Besides, there are great individual differences among subjects. Supplementation of specific bacterial strains or prebiotics has its limitations and may not benefit all individuals. Thus, it is of interest to develop novel methods that precisely regulate gut microbiota.

### 3.2 GMMs and ISCs

Gut microbiota is capable of biotransforming either host-derived components such as bile acids or dietary substances to generate various metabolites. Several studies have revealed that GMMs had effects on the host's gut environment as well as the immunological balance.

#### 3.2.1 Short-chain fatty acids

Short-chain fatty acids (SCFAs) as the fermentation products of indigestible carbohydrates, primarily comprising acetate, propionate, and butyrate, are the most studied metabolites of gut bacteria 53. SCFAs are important energy source of colonocytes. Besides, they directly or indirectly impact on host physiology, as some of SCFAs also serve as the substrate and regulator for intestinal and hepatic gluconeogenesis.

The role of SCFAs on ISCs has been intensively investigated. Since the effect of SCFAs on ISC is confounded by multiple factors, such as diet, gut microbiota, and gut physiology, opposing conclusions have been drawn from recent decades of research 54. One part of the studies suggests that SCFAs have a proliferative effect on ISC, while conclusion from the other part supports an ineffective or even an inhibitory effect.

In earlier years, Sakata* et al.* found that SCFAs stimulate EC proliferation through the autonomic nervous system in association with dosage of SCFAs, the uptake of intestinal microorganisms and fermentable fibers 55, 56. *In vitro* experiments have demonstrated that SCFAs promoted mucosal proliferation 57, 58. It is reported that, in small intestinal organoids, HDAC inhibition by butyrate resulted in an enhanced LGR5^+^ ISC pool 59. Donohoe *et al.* suggest that microbial production of butyrate, the main source of energy for colon cells, may provide energy for ISCs and promote its value-added 60. De Vadder *et al.* demonstrated that SCFAs promoted intestinal gluconeogenesis through a cAMP-dependent mechanism to control glucose metabolism 61. Therefore, these studies have suggested a promotive role of SCFAs on ISC proliferation.

However, Boffa *et al.* illustrated that feeding wheat bran significantly reduced the level of butyrate in the colon and inhibited the proliferation of colon cells 62. Kaiko and colleagues showed that butyrate inhibited the proliferation of ISCs upon mucosal injury or in crypt-less host organism 63. In addition, Scheppach demonstrated that butyrate has no effect on the growth of crypt cells 64.

**Figure 4:**
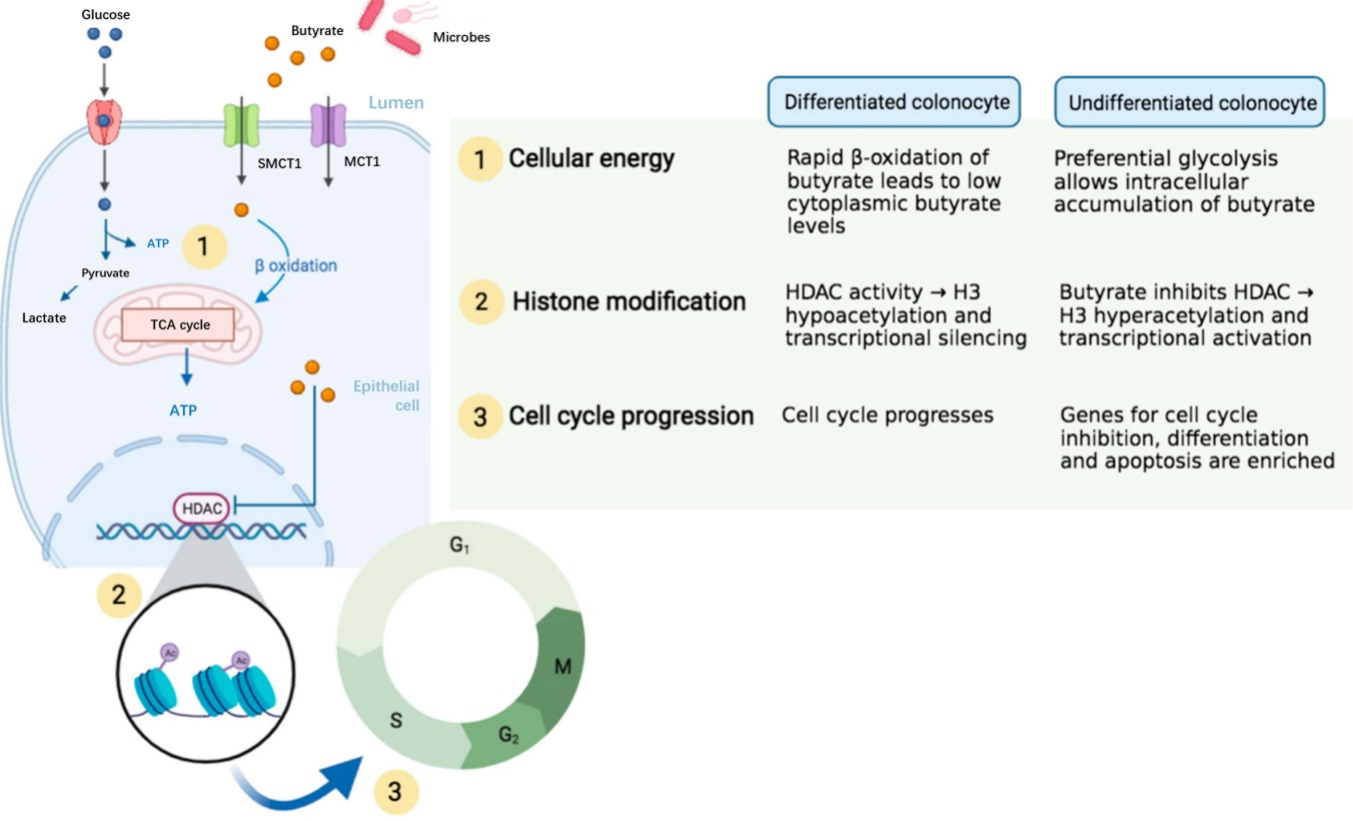
** A proposed epigenetic configuration involving distinct metabolic pathways that contributes to “butyrate paradox”.** Figure reused with permission from Ref. 65. Copyright @ 2021 by Pooja S. Salvi and Robert A. Cowles.

Despite the disagreement regarding the role of SCFAs on ISCs, most of studies have indicated that SCFAs particularly the butyrate enhanced ISC proliferation. The difference in experimental design and conditions across research groups may contribute to the discrepancy of results. For the *in vivo* studies, the gut microbiota of animals in different houses may be varied in response to dietary fibers or SCFAs. For the* in vitro* studies, the concentration and culture conditions used may significantly lead to bias in results. In addition, SCFAs have diverse effects on ISCs through not only influencing ISC proliferation but also modulating genetic pathways that might or might not be associated with ISC proliferation. Some researchers have proposed that the distinct metabolic pathways for energy use associating with epigenetics in differentiated and the undifferentiated colonocytes may contribute to the “butyrate paradox” (Figure 4) 65. Therefore, more studies are advocated to investigate the exact role of SCFAs on ISCs.

Besides the SCFAs, several studies have demonstrated that SCFA intermediate metabolites such as lactate and succinate are regulators of ISCs. Fevr *et al.* showed that lactate led to larger size of small intestine organoids and enhanced expression of target genes in Wnt/β-catenin pathway which modulates stem cell self-renewal 66, 67. On the contrary, succinate is reported to mediated superoxide production and mucosa damage in the colon 68. It is showed that succinate through colonic infusion inhibited colonic epithelial growth in rats 69.

#### 3.2.2 Tryptophan metabolites

Tryptophan as an essential amino acid is readily metabolized by microorganisms to a variety of biologically active amino acid derivatives, such as indole, indoleacetic acid (IAA), indole aldehyde (IAld), indole acrylic acid (IA), indole lactic acid, indole ethanol, indole propionic acid, tryptamine, 3-methylindole, *etc.* 70-73. These metabolites are mostly present as ligands for the aryl hydrocarbon receptor (AhR) and the pregnane X receptor (PXR) 74-76. Although both AhR and PXR participate in immune regulation, intestinal barrier function and cell proliferation, current knowledge mainly supports a role of the microbial metabolites of indole derivatives on ISC via AhR signaling. How tryptophan metabolites influence ISC through PXR remains further studies.

Intestinal microbe-derived indole metabolites influence ISCs through acting as ligand of AhR. It is reported that the expression level of β-catenin was unexpectedly high in the cecum of *Ahr*^‑/-^ mice, which was decreased after treatment of IAA. IAA suppressed β-catenin signals through AhR-mediated β-catenin degradation 77. The stem cell niche is maintained by *Rnf43* and *Znrf3* by blocking Wnt/catenin signaling and limiting its proliferation, and the gut barrier is restored by AhR signals in response to dietary ligands 78. Recently, Metidji *et al.* reported that indole-3-carbinol (I3C) inhibited ISC proliferation through activating AhR, which led to the increased expression of *Znfr3* and *Rnf43* genes for degrading Wnt 79. In addition, Park *et al.* also showed that I3C could inhibit ISC proliferation 80.

The microbial indole metabolites also affect ISCs activity through interaction indirectly with other cells. Wlodarska showed that oral administration of* Peptostreptococcus russellii* to mice resulted in increased production of IA which activated AhR in colonic organoids and macrophage and contributed to elevated expression of Ki67 and proliferation of goblet cells in DSS-induced colitic mice 81. Previous studies also demonstrated that *Lactobacillus reuteri* enhanced mouse intestinal EC proliferation through generating IAld metabolite which activated AhR in lamina propria lymphocytes to upregulate IL22 and promote ISC proliferation 82, 83.

#### 3.2.3 Secondary bile acids

Since high levels of diet-induced secondary bile acids (SBAs), such as lithocholic acid (LCA) and deoxycholic acid (DCA), may contribute to the development of colorectal cancer, it can be expected that SBAs have a role in the proliferation of ISCs 54, 84. The direct evidence has been limited. Kozoni *et al.* reported that LCA increased proliferating cells in colonic crypt of mice 85. Other findings in cancer cells showed that DCA increased Wnt/β-catenin signaling and promoted colon cancer cell proliferation at low concentrations. However, the effect was reversed at high concentrations 86, 87. This phenomenon was also reported by Dossa and Milovic's works 87, 88. These results may suggest a potential role of SBAs on ISCs. However, till now, the direct effect of SBAs on ISCs both *in vitro* and *in vivo* is not fully understood.

#### 3.2.4 Other microbial metabolites

A study by Jones showed that *Lactobacillus plantarum* caused alterations in gut epithelial cell growth by promoting physiological amounts of reactive oxygen species (ROS) from Nox enzyme 89. In 2018, Latsenko *et al.* reported that a zero mutation in the Drosophila PGRP-SD gene was related to increased overgrowth of *L. plantarum* and its metabolite lactate through activation and generation of ROS 90. In the same year, Lee and colleagues demonstrated that GPR81 on panthenol and stromal cells recognized lactic acid derived from *lactobacilli* and induced regeneration of intestinal ECs via WNT3/β-catenin 91. Reedy *et al*. then corroborated the findings of the aforementioned experiments 92.

Polyamines are a type of compounds that can be either from dietary source or produced by gut microbiota. It was reported that polyamines may serve as energy source or influence intracellular signaling pathways such as the TGF-βRI/Smad3/4, c-fos and c-jun, *etc.* to promote ISCs proliferation 93-95.

### 3.3 Dietary influence

Dietary variables may have a direct impact on gut homeostasis either directly or indirectly through regulating the composition of the intestinal microbiota and/or microbial metabolites.

Frieling and colleagues have found that HFD increased ISCs activity in the Drosophila fruit fly. Specifically, the intestinal microbiota and the JNK signaling pathway in intestinal cells were both required for this action to occur 96. Hou *et al.* identified diet-microbial metabolism feedforward loop that showed a remarkable role in modulating ISC proliferation in the stressed gut. They found that dietary raffinose promoted *Lactobacillus reuteri* growth which increased metabolism of raffinose to fructose for enhancing glycolysis to support ISC proliferation and renewal 97.

Gut microbiota mediated dietary metabolites showed potential effects on ISCs. Singh *et al.* demonstrated that urolithin A, a major microbial metabolite of phenolics from berries and pomegranate fruits, exerted barrier activity by upregulating epithelial TJ proteins in intestinal ECs via the AhR-nuclear factor erythroid 2-related factor 2 (Nrf2) pathway in organoid culture 98, suggesting a potential role in regulating ISCs.

## 4. Signaling pathways associated with regulation of ISCs

### 4.1 Notch signaling pathway

**Figure 5:**
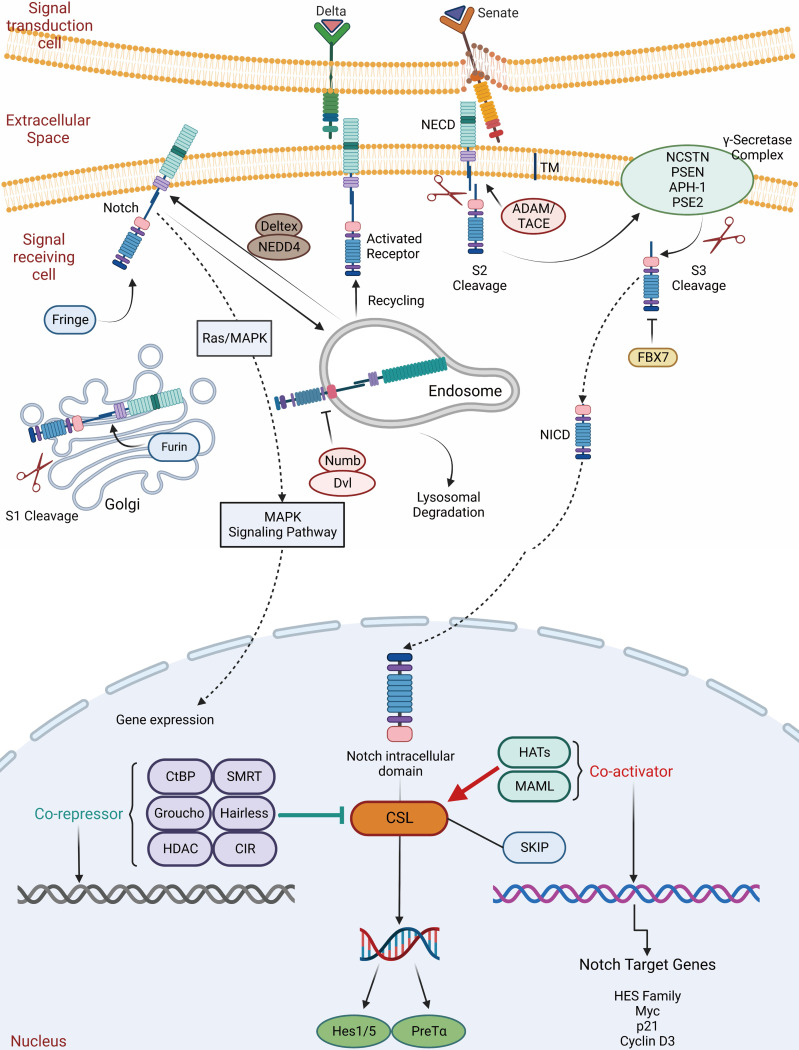
** The Notch signaling pathway in ISC regulation.** The Notch signaling receptor is a single transmembrane protein with functional extracellular (NECD), transmembrane (TM) and intracellular (NICD) structural domains. Delta and Senate families are ligands for Notch. First, the Notch receptor is sheared and glycosylated within the Golgi of the recipient cell, followed by S1 cleavage with TM-NICD. Endosomes transfer processed receptors to the plasma membrane, where it binds to the ligand via Deltex regulation and NUMB inhibition. Secondly, TACE (TNF-ADAM: metalloproteinase convertase) shears NECD away from the TM-NICD structural domain when it binds to the ligand (S2 cleavage). Lastly, NECD-linked ligands form a complex. In signal-receiving cells, NICD is released from the TM by-secretase (S3 cleavage), resulting in nuclear translocation, binding to the CSL transcription factor complex and allowing transcription of traditional Notch target genes.

During development and in adult tissues homeostasis as well, the Notch pathway plays an essential role in determining cell fate, which is a highly conserved mechanism. The Notch system regulates pro-secretory cell signaling by influencing both signaling and receiving cells through ligand-receptor interactions. The Notch signaling receptor is a single transmembrane protein with functional extracellular (NECD), transmembrane (TM) and intracellular (NICD) structural domains. Delta-like and Senate (Jagged1, Jagged2) families are ligands for Notch. First, the Notch receptor is sheared and glycosylated within the Golgi of the recipient cell, followed by S1 cleavage with TM-NICD. Endosomes transfer processed receptors to the plasma membrane, where it binds to the ligand via Deltex regulation and NUMB inhibition. Secondly, TACE (TNF-ADAM: metalloproteinase convertase) shears NECD away from the TM-NICD structural domain when it binds to the ligand (S2 cleavage). Lastly, NECD-linked ligands form a complex that is dependent on endocytosis and cycling via Mib ubiquitination in signaling cells. In signal-receiving cells, NICD is released from the TM by-secretase (S3 cleavage), resulting in nuclear translocation and binding to the CSL transcription factor complex, allowing transcription of traditional Notch target genes (Myc, p21, and HES family members) (Figure 5) 99-103.

Notch signaling includes Notch receptors, CSL DNA binding proteins, and Notch ligands (DSL proteins), which determines biological cell development by regulating local intercellular interactions and are essential for the maintenance of ISCs 104. Researchers found that activation of Notch signaling increased ISCs while inhibiting cell differentiation, suggesting that the Notch pathway regulates early intestinal cells 105. Notch, in contrast to other receptors, receives signals from the surface of the cell and regulates gene expression inside the nucleus. Notch signaling regulates intestinal cell types, and ISCs differentiate into two types: secretory intestinal enteroendocrine cells and nutrient-absorbing intestinal ECs 106. Ectopic overexpression of NICD decreased the frequency of secretory cells in the mouse intestinal epithelium 107-109. Inhibiting the Notch pathway, on the other hand, resulted in increased secretory cell lines and fewer absorptive enterocytes and colitis cells 106, 110-112. Guo and Ohlstein also demonstrated that ISCs with high Delta levels strongly activated Notch signaling in their progeny, resulting in ECs, whereas Notch signaling inhibition or loss resulted in stem cell loss and premature formation and appreciation of enteroendocfine cells; thus, the regulation of Notch signaling on ISC differentiation is bidirectional 113. To regulate ISC behavior, activity oscillates within a limited threshold. Increased Notch activity promotes ISC development, resulting in the creation of more daughter cells, with increased Notch activity leading to intestinal stem cell differentiation 114. These data indicate that Notch signaling is crucial to determining how intestinal epithelium develops 13, 108, 114-117.

Recent research has indicated that Notch's influence on the ISC spectrum was mediated through its action on *Atoh1*, with *Hes1* and *Atoh1* expression being mutually inhibited 118. Thus, the degree of Notch activity controls the reciprocal regulation of *Hes1* and *Atoh1*, which is critical for balancing the destiny of cells between uptake and secretion 119, 120. Furthermore, Atoh1 is controlled by Wnt signaling, whose activity affects GSK3β, a protein kinase that mediates phosphorylation and protein degradation and regulates *Atoh1* via ubiquitination 120-122. Moreover, *Gfi1*, a transcription factor that is reliant on *Atoh1* expression in the intestine, is essential for the differentiation of *Atoh1*-specific secretory precursors into enteroendocrine and cupped/pan precursors. An investigation has shown that defective *Neurog3* suppression at an early stage was required for sub-stasis to occur in Paneth and mucus cell lines, and that sub-stasis resulted in alterations in the cell type ratio in *Gif1*-deficient mice. Thus, analogous to *Hes1*/*Atoh1*, the relative activity of *Gfi1*/*Neurog3* is implicated in the partitioning of cells into secretory lineages 123.

ISC self-renewal is maintained and modulated by Notch signaling. Kapuria *et al.* found that inhibition of Notch-mediated Tuberous Sclerosis Complex 2 (TSC2) helped to promote the differentiation of ECs 124. Qu *et al.* revealed that dual cortisol-like kinase 1 (Dclk1) may act as ISCs marker and that restricting the Notch signaling pathway decreased ISC survival, implying that the Notch pathway is critical for ISC-mediated saphenous fossa regeneration. Dclk1 expression in saphenous ECs may potentially be used as a measure of ISC survival after injury 125. Bmi1 expression is dynamically controlled and depends on synergistic regulation by Notch, MaM co-activators and β-catenin. Bmi1 regulates mouse ISC proliferation and self-renewal downstream of Notch 126. Srinivasan *et al.* then proved that Notch signaling modulated the Bmi1/Lgr5^+^ ISCs ratio, with suppression of Notch signaling decreasing this ratio and vice versa increasing this ratio. This confirms that Notch signaling can induce asymmetric division in response to intestinal inflammation, establishing a direct link between slow and fast-cycle ISCs 127. Kwak *et al.* observed that Ghrelin rehabilitates gut function after intestinal lesions by activating Notch signaling 128. Jones *et al.* established that Defacre-expressing Paneth Cells were malleable and that the Notch signaling pathway may induce differentiation of Defacre-expressing Paneth Cells into pluripotent stem cells expressing Lgr5^+^ crypt base columnar and facilitated acute intestinal regeneration following damage 129.

Recently, the laboratory of Joshua V. Troll demonstrated for the first time that the MyD88-dependent signal induced by the microflora inhibited the Notch signal, thereby promoting the fate of secretory cells. These results link the activity of the microflora with the Notch pathway. In conclusion, Notch signaling is essential for intestinal homeostasis and repair, which when damaged may lead to chronic inflammation and cancer 130.

### 4.2 Wnt signaling pathway

**Figure 6:**
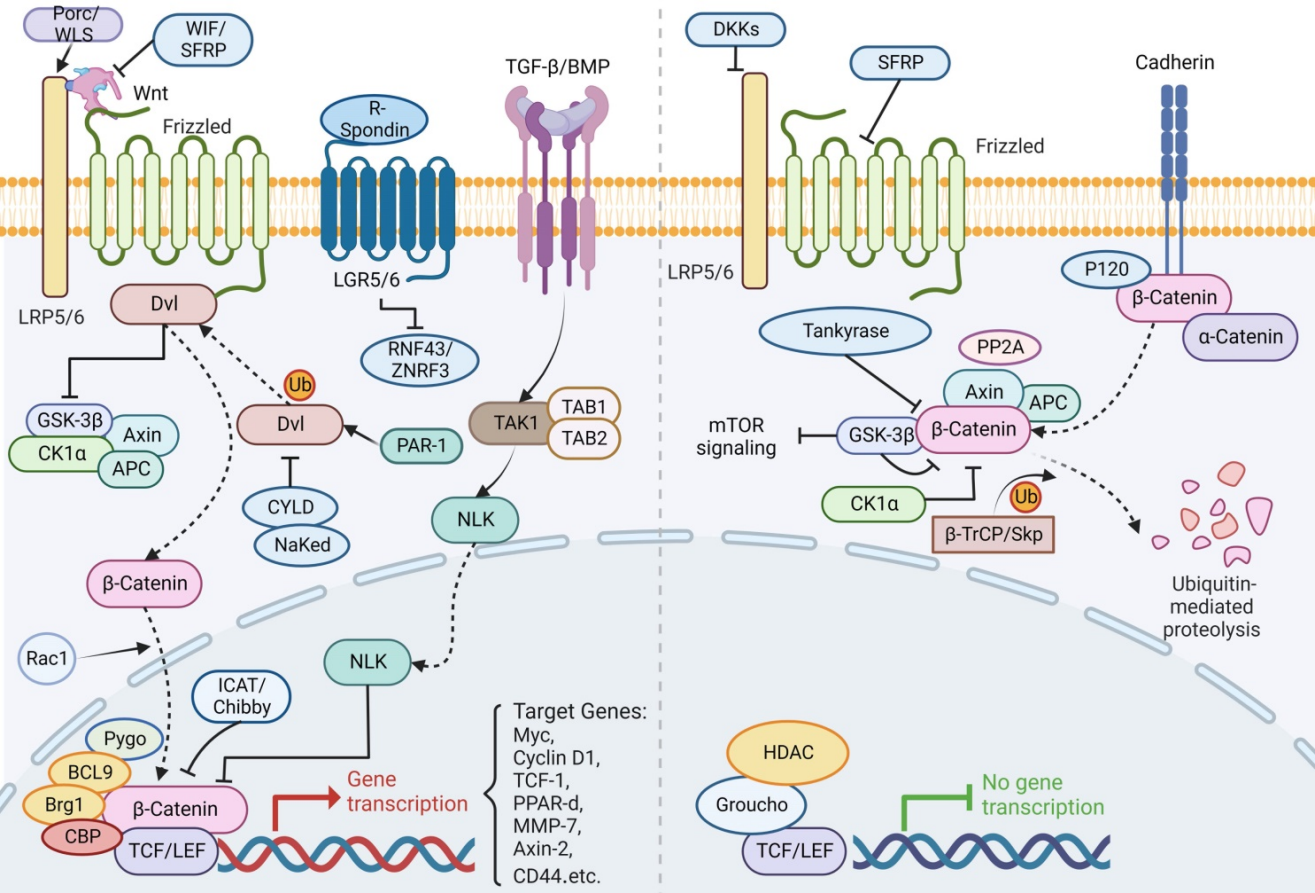
** The canonical Wnt/β-catenin signaling pathway in ISC regulation.** A ligand for the canonical Wnt/β-catenin attaches to Frizzled receptors then assembles a complex on the surface of the cell together with LRP5/6. The binding of R-spondin to LGR5/6 inhibits the frizzled receptor's activity by ubiquitinating ZNRF3 and RNF43. When the Wnt receptor complex is activated, GSK-3β is dissociated from APC/Axin/GSK-3β. Dvl is activated by sequential phosphorylation, polymerization and polyubiquitination in response to PAR-1 and is inhibited by CYLD and NaKed. Stable β-catenin enters the nucleus through Rac1, contacts LEF/TCF, displaces co-repressors, then recruits co-activators in Wnt target genes. On the other hand, TGF-β/BMP regulates NLK into the nucleus through TAK1 to inhibit β-catenin expression. Without Wnt signaling, β-catenin acts as a transduction co-regulatory molecule and an intercellular adhesion junction protein, which is phosphorylated by CK1α and the APC/Axin/GSK-3β complex, resulting in ubiquitination and degradation by the proteasome via the β-TrCP/Skp pathway. In addition, β-catenin works in concert with several different transcription factors to control specific targets.

The Wnt pathway is crucial to early development, organogenesis, tissue regeneration and other physiological processes in the animal embryo, which is a highly conserved class of signaling pathway. It's a collection of several downstream signaling pathways that are activated when the ligand protein Wnt binds to membrane protein receptors. The Wnt signaling pathway enables two distinct modes of communication between cells: intercellular (paracrine) communication and autophagic communication (autocrine). The canonical pathway is comprised of Dvl, GSK3, the Wnt family of secretory proteins, APC, Axin, the Frizzled family of transmembrane receptor proteins, β-catenin, and TCF/LEF transcriptional regulators. The planar cell polarity route is involved in cytoskeletal rearrangement and JNK activation; the Wnt/Ca+ pathway is involved in triggering PLC and PKC; additionally, the intracellular pathway is involved in spindle orientation and asymmetric cell division 131. The following section will discuss the canonical Wnt/β-catenin pathway in further detail. It regulates ISC pluripotency and determines the differentiation fate of cells throughout development. Moreover, it incorporates signals including retinoic acid, fibroblast growth factor (FGF), transforming growth factor beta (TGF-β) and BMP. A ligand for Wnt attaches to Frizzled receptors then assembles a complex on the surface of the cell together with LRP5/6. The binding of R-spondin to LGR5/6 inhibits the frizzled receptor's activity by ubiquitinating ZNRF3 and RNF43. When the Wnt receptor complex is activated, GSK-3β is dissociated from APC/Axin/GSK-3β. Dvl is activated by sequential phosphorylation, polymerization and polyubiquitination in response to PAR-1 and is inhibited by CYLD and NaKed. Stable β-catenin enters the nucleus through Rac1, contacts LEF/TCF, displaces co-repressors, then recruits co-activators in Wnt target genes. Without Wnt signaling, β-catenin acts as a transduction co-regulatory molecule and an intercellular adhesion junction protein, which is phosphorylated by CK1α and the APC/Axin/GSK-3β complex, resulting in ubiquitination and degradation by the proteasome via the β-TrCP/Skp pathway. In addition, β-catenin works in concert with several different transcription factors to control specific targets (Figure 6) 132-141.

Not only is Wnt the main force in ISC proliferation, but its mutagenesis is also a major factor in colon cancer 4, 142. Its activity contributes to intestinal differentiation as well as the stability of ISCs, and either an increase or decrease in β-catenin activity has a significant effect on intestinal epithelial cell generation and differentiation, ultimately leading to the development of GI tumors 143. Numerous studies have indicated that mutations that activate the Wnt pathway abnormally could accelerate the proliferation of undifferentiated progenitor cells and ultimately resulted in cancer 144. Wnt/β-catenin signaling activation is often related to carcinogenesis, most notably in colorectal cancer. Zhang *et al.* revealed a previously unknown method of β-catenin activation, the MST4-p-catenin signaling axis. A MST4 deficiency led to a reduction of ISCs and slowed colon cancer formation. Secondly, the MST4-p-catenin axis was elevated, which has been associated with a poor outcome in human colorectal cancer. In conclusion, this study broadens avenues for colon cancer targeted treatment 145.

Several studies have found that changes in the Wnt pathway and microbial composition regulated the proliferation of intestinal ECs through Myd88 146-148. Hirohito Abo *et al.* showed that early microbiota regulation of Erdr1 stimulated the Wnt pathway in ECs, boosted Lgr5^+^ stem cell production, and promotes colonic mucosal repair, which may be utilized to treat cancer and mucosal ulcers 149.

Nevertheless, several protein ligands and cytokines have the ability to regulate the Wnt signaling. Strubberg *et al.* noted that inhibition of proliferation of ISC in patients with cystic fibrosis (CF) by cystic fibrosis transmembrane conductance regulator (Cftr) may promote Wnt/β-catenin signaling, thereby increasing the risk of gut neoplasia 150. Johansson *et al.* proved that RALs are required for effective ISC regeneration downstream of Wnt signaling. ISC function and Lgr5 positivity decreased, resulting in fast crypt mortality and insensitivity to Wnt inhibition, and impaired tissue regeneration 151. Han *et al.* reported that YTHDF1, which is distinctly expressed in ISCs, enhanced β-catenin activity by increasing the translation of Wnt signaling. It contributes to the maintenance of ISCs during regeneration and tumorigenesis 152. Moreover, Li *et al.* proved that deletion of Lats1/2 (the core Hippo kinase) inhibited the Wnt pathway, leading to deficiency of ISC, which is based on transcriptional enhanced associate domain (TEAD). Their team recognized that inhibition of TEAD palmitoylation could prevent the above finding 153. Wei *et al.* showed that Erk1/2 deficiency resulted in activation of the Ras/Raf cascade through activation of Wnt signaling, which then transduced Akt activity to enhance Wnt/β-catenin pathways as well as the mTOR. This was associated with a decrease in mesenchymal Bmp4, which is a Wnt repressor 154.

To effectively treat chronic intestinal illnesses, it's necessary to completely understand the developmental mechanisms of ISC. However, there's a dispute about the various ISC lineage hierarchy and segregation theories. Anika Böttcher and colleagues demonstrated a new ecological signal Wnt/PCP pathway in the development of ISC 155. While Wnt signaling governs ISC self-renewal by inducing Lgr5^+^, little is known about Lgr5 post-translational regulation. Novellasdemunt *et al.* indicated that silencing NEDD4 and NEDD4L increased Wnt activation and the amount of ISCs, hence enhancing tumor predisposition and progression 156.

In addition, a study found that increased zinc activity maintained gut integrity through activation of Wnt/β-catenin signaling, shedding insight into the efficient preventative approach of ISC-based exogenous zinc preparations 157. Dectin-1 (a C-type lectin receptor) could signal to intestinal ECs to activate the Wnt pathway and induced intestinal ECs to proliferate, thereby promoting colorectal cancer (CRC) development 158.

### 4.3 BMP signaling pathway

**Figure 7:**
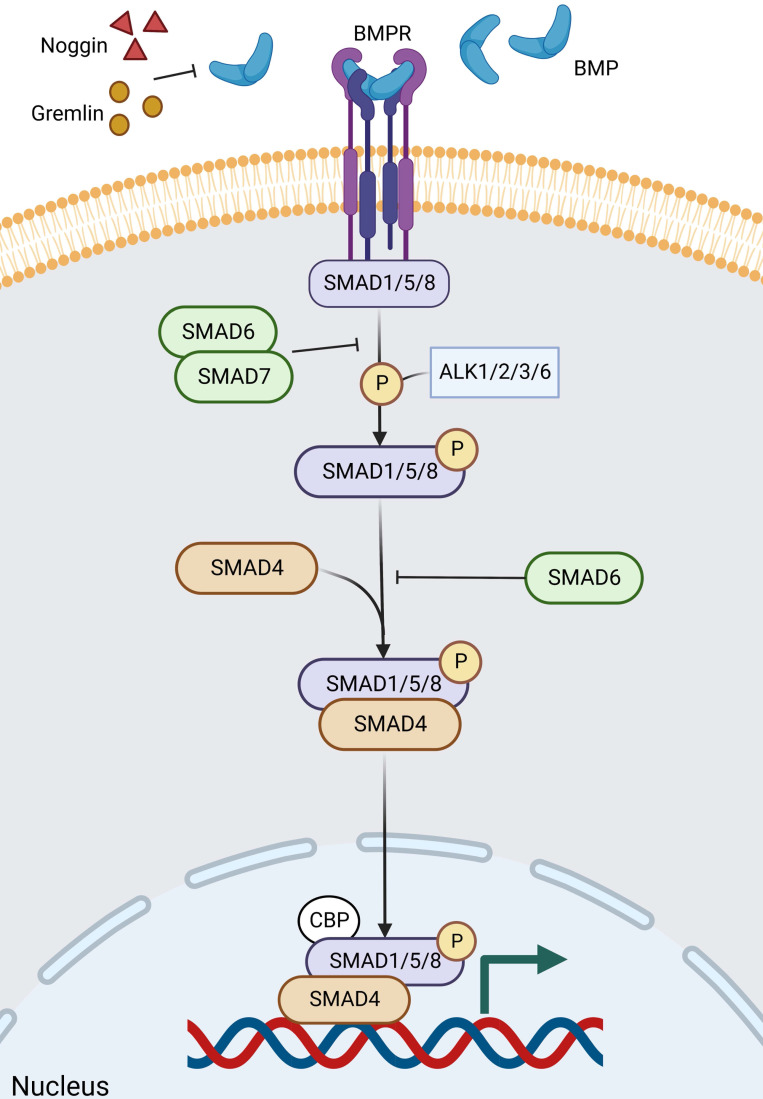
** The BMP signaling pathway in ISC regulation.** BMP signaling contains a complex formation of different type 1 and type 2 receptors for serine/threonine kinase activity. Noggin and Gremlin are extracellular inhibitors of BMP. After ligand binding to the receptor and entry into the cell, firstly, the downstream molecules of BMP signaling, SMAD1/5/8, are phosphorylated by ALK2/3/6 and ALK1, and then BMP signaling is highly activated by the phosphorylated SMAD1/5/8. Of these, SMAD6/7, particularly SMAD6, is involved in inhibiting phosphorylation and signaling, which inhibits ALK3/6-mediated signaling. Finally, phosphorylated SMAD1/5/8 aggregates with Smad4 and continues to signal, accumulating in the nucleus and regulating target gene transcription.

BMP is a growth factor of the TGF-β family, and BMP signaling contains a complex formation of different type 1 and type 2 receptors for serine/threonine kinase activity. Ligand recognition can be obtained from both type 1 and type 2 receptors 159. During epithelial cell differentiation, SMAD1/5/8, a molecule downstream of BMP signaling, is phosphorylated by ALK2/3/6 and ALK1, followed by BMP signaling highly activated by phosphorylated SMAD1/5/8 160, 161. SMAD6/7 is involved in the inhibition of phosphorylation and signaling, particularly SMAD6, which inhibits ALK3/6-mediated signaling (Figure 7) 161, 162. The main BMPs (both BMP2 and BMP4) in the gut are recognized by the type 1 receptor BMPR1A, which is expressed in mature epithelial cells and mesenchymal cells 163-165. BMP is negatively regulated by ISCs in the gut 166, 167, and its inhibitors, Noggin and Gremlin (Grem1 and Grem2) are expressed in the crypt 164, not only keeping ISCs and progenitor cells in a BMP-low niche 166, 168-172, but also inducing stem cell proliferation and enhancing Wnt activity 168. Among other things, there is a negative crosstalk between Wnt and BMP/TGF-β 165.

It has been shown that BMPR1A and SMAD4 mutations cause the human polyposis. If BMPR1A is deleted or overexpressed in mice, ectopic crypt formation is stimulated 161, 164, 168. In addition, abnormalities in Grem1 or Noggin can also lead to human polyposis, which is related to mutations in BMP pathway genes and a high-risk propensity for CRC 173, 174. Mechanistically, SMAD/HDAC1-mediated repression of stem cell-associated genes (e.g. Lgr5, Sox9) is driven by BMP signaling, which prevents premature ISC expansion and polyp formation in Wnt-rich niche 175. Recently, a study has revealed how oncogenes convert the niche environment into a beneficial one - niche remodeling. Secretion of BMP ligands by crypt expressing oncogenic KRAS or PI3K, inhibition of ISC activity, and alteration of Wnt signaling by PDGFRloCD81^+^ stromal cells induced by crypt with oncogenic PI3K 176. In conclusion, BMP signaling sustains homeostatic balance by suppressing Lgr5^+^ ISC proliferation at the base of the crypt while promoting the allocation of secretory cell lines 177.

### 4.4 Other pathways

Likewise, other pathways and factors affect intestinal homeostasis and the progression of GI cancers. JAK-STAT signaling has been proven to control the proliferation of ISC and is negatively regulated by Notch 178. A crucial question is how the intestinal epithelium interacts with the bacterial flora and microbial metabolites and what the mechanism is at the molecular level. Smarcad1 is a member of the Smarcad family of chromatin remodeling factors and is highly expressed in the epithelial region of the gut. Kazakevych and colleagues discovered that Smarcad1 mediated microbially caused inflammatory reactions in mice and coordinated the expression of intestinal epithelial cell gene transcription 179.

Vitamin D/vitamin D receptor (VDR) inadequacy is a major risk factor for colon cancer. Zhang *et al.* postulated that the intestinal VDR protected mice against dysbiosis by modulating the JAK/STAT system. VDR insufficiency is a major risk factor for colon cancer. They identified potential targets for cancer prevention, the VDR, which protected against the effects of dysbiosis by regulating the JAK/STAT system 180. TNFAIP8 is a regulator of the Akt/β-catenin signaling axis for intestinal injury repair, which regulates the homeostasis and regeneration of mouse ISC through modulation of microbiota-induced Akt signaling pathways 181.

In addition, YAP1 activity is implicated in the maintenance of ISCs proliferation which acts as an inhibitor of Hippo pathway downstream effectors and/or Wnt signaling, contributing to ISC maintenance and differentiation in transit-amplified cells. The structural integrity of intestinal EC junctions is the primary signal regulating intestinal homeostasis via the Hippo pathway 182. A recent study demonstrated that suppression of Hippo signaling reprogramed Lgr5^+^ ISCs to a *Klf6*^+^ wound-healing cell state, and YAP overexpression led to inhibition of tumor formation 148.

Finally, autophagy and its regulatory mechanisms are involved in the homeostasis and repair of the gut by regulating TJs and influencing the metabolism and the proliferative and regenerative capacity of ISCs 183. Defective Atg16l1 autophagy in intestinal ECs led to abnormal morphology of Paneth cells, as evidenced by dysfunction in the secretion of protective antimicrobial peptides 184-186, and resulted in impaired ISC capacity as well 187. This was because ISCs relied on Wnt and EGF signals to maintain stemness, and these signals are partially provided by Paneth cells. In addition, autophagy-deficient ISCs were more susceptible to excess ROS 188, 189, which made repair of intestinal damage much more difficult. Besides, it was found that deletion of Atg7 led to increased oxidative stress, which promoted P53-mediated apoptosis in Lgr5^+^ ISCs 190.

## 5. Current development of intestinal organoid culture

In 2009, Sato *et al.* successfully cultured crypt extracts from the murine small intestine into self-renewing and differentiating organoids 25. The successful establishment of the first miniature intestinal organoid opens a new chapter in organoid research and is rapidly becoming a new research hotspot. The intestinal organoid is three-dimensional (3D) cell culture grown from pure ISCs. In 3D culture systems, these organoids can provide an *in vitro* model that more closely resembles the *in vivo* environment than traditional two-dimensional (2D) cell culture technique. ISCs replicate the self-renewal seen* in vivo* in the mature gut, resulting in forming a crypt-villi structure, epithelial polarization and functional lumen 25, 191, 192.

The 3D organoids have many applications in disease modeling, gene function analysis, and regenerative medicine. UC is characterized by chronic inflammation in the colon, and prolonged exposure to an inflammatory environment predisposes to CRC 193. To understand the genetic mechanisms of inflammation, Nanki *et al.* analyzed somatic mutations in the epithelium of UC using organoids 194. In the same year, a parallel study sequenced UC and non-UC crypt foci isolated directly from patients and reported frequent mutations in UC on the NFKBIZ pathway 195. Pleguezuelos-Manzano *et al.* showed by organoid that pks^+^ *E. coli* induced double-stranded DNA breaks and interstrand crosslinks, and that individuals carrying genotoxic pks^+^ *E. coli* individuals may be at higher risk of developing CRC 196. Notably, several studies showed that transplanted Lgr5^+^ stem cells led to recovery of damaged colonic lesions and the repaired colonic structures displays appropriate epithelial barrier function 197.

Although organoids have been vastly superior to normal 2D cell cultures, there are still drawbacks, such as a lack of endothelial cells, immune cells and gut microbes, which limits the study of cell-cell and cell-microbe interactions. To better study the interaction of intestinal cells with other types of cells or gut microbe, the recent development of a human intestinal microfluidic organ-on-a-chip model has been engaged to overcome these challenges. Organ chips can integrate different types of cells including ECs, microvascular endothelium, nerve cells, immune cells as well as their interplay with gut microbes 198-200. Some studies have used immortalized cell lines such as Caco-2 to generate gut chip 201, 202. However, these cell lines are usually originated from tumor origin and are not suitable for studying intestinal tumorigenesis and normal physiology. The emerging primary culture based on combing organoid and organ chip has made much progress, which enables fluid flow and peristalsis-like deformations and allows the intestinal ECs undergo villus histogenesis with multilineage differentiation that mimics the growth and host defense response of original tissue 203, 204.

As the 3D organoids have enclosed lumens that may limit their value for transport and coculture studies, recently the microinjection technique shows some advantages. For instance, microinjection of fecal microbes into colon organoids demonstrated that the complex microbiota in feces survived and grew in the colon with little change in complexity 205, 206.

In addition, Meran *et al.* constructed autologous jejunal mucosal grafts using biomaterials, which showed that this biological scaffold can be efficiently expanded *in vitro* and has highly similar biochemical properties to those *in vivo*, becoming a boon for patients with intestinal failure 207. The current shortcomings and problems with this technology include the difficulty of constructing some macroscopic organ functions, technical robustness, production materials, *etc.* 198.

## 6. Conclusions and future directions

The digestive tract has an exceptional capacity for homeostasis, as demonstrated by the efficient absorption and secretion of nutrients. As well, it has a strong mucosal barrier that prevents the invasion of pathogenic bacteria and the excessive renewal of epithelial cells and tumor overgrowth, and generates anti-inflammatory components such as IgA and antimicrobial peptides against GI inflammation. Numerous GI and immunometabolic diseases are closely linked to disturbances in gut homeostasis; therefore, intestinal ECs must repair themselves after various pathogenic lesions. The renewal of the intestinal ECs depends on the regulation and support of the ISC and stem cell niches. To maintain dynamic stability, the niche needs to be continuously reshaped and adapted. Notably, the maintenance of ISC activity, which is tightly regulated by several signaling pathways, is influenced by inflammation, gut microbiota, and GMMs.

Numerous evidence has demonstrated that gut microbiota or their GMMs help maintain gut health during homeostasis and, of particular note, they play a critical role in regulating ISC activity to protect against injury. Specific bacteria (*e.g.*, *Bacillus* and *Lactobacillus*) and GMMs (*e.g.*, SCFAs) have been identified to directly influence ISC proliferation, renewal and differentiation. Given that the gut microbiota is comprised of a large community of microbes that can be either healthy or pathogenic, it is assumed that the interaction between gut microbes and ISCs is still largely unknown. Thus, a critical future direction for the field of ISCs is to investigate the variables and processes underlying the interaction between the intestinal microbiota and the intestinal epithelium, and more precisely, which signaling pathways or ligand factors are involved, by utilizing finer and more precise molecular markers.

It should be noted that gut microbiota may indirectly impact the stem cell niche via modulating other cells to regulate ISCs. A latest study revealed complex regulation between the gut microbiota, gut neurons, immune cells and ISCs 208. The metabolite valeric acid from the gut microbiota promoted Tph2 expression in intestinal serotonergic neurons by blocking recruitment of the NuRD complex to the Tph2 promoter. The 5-hydroxytryptamine activated PGE2 production in the PGE2 macrophage subpopulation through its receptor HTR2A/3 A, and PGE2 promoted Wnt/β-catenin signaling by binding to EP1/EP4 to promote ISC self-renewal. A new level of ISC regulation by niche cells and gut microbiota is uncovered 208. Therefore, it is essential to explore the complex interactions among gut microbiota, niche cells and ISCs.

In addition, the proper functioning of ISC is dependent on the influence of various signaling pathways such as Wnt, Notch, BMP, autophagy and so on. For example, the Notch pathway plays an influential role in determining cell fate. Wnt/β-catenin activity contributes to ISC differentiation as well as stability, and mutations that aberrantly activate the Wnt pathway may accelerate the proliferation of undifferentiated progenitor cells, ultimately leading to cancer. In contrast, BMP signaling inhibits the proliferation of Lgr5^+^ ISC at the base of the crypt. Defects in autophagy-related genes not only affect the proliferation and differentiation of ISC, but also cause abnormal secretion of antimicrobial peptides by Paneth cells, leading to diseases with impaired intestinal barrier. Other pathways such as YAP, VDR and JAK/STAT also regulate ISC activity. Indeed, in order to understand the role of ISCs in gut homeostasis, it is essential to investigating the tight regulation of ISC maintenance, proliferation, differentiation and stability.

Further, most previous studies on ISCs used drosophila or mouse models of the gut. However, in recent years, there has been a growing interest in human intestinal organoids, achieving an increasing number of exciting achievements. The interaction between ISC and other type of cells or gut microbe could be explored. The new development in primary human organoid culture and organ chip will largely facilitate basic research in precision medicine, bridging the gap between traditional *in vitro* high-throughput screening and *in vivo* studies used in disease modeling and drug development, and has potential for regenerative medicine research.

## Figures and Tables

**Table 1:** Alteration of gut microbiota in diseases and the associated mechanisms.

Disease	Characteristics or Alterations	Effects	References
Healthy subject	High gut bacterial diversity and microbial growth rates; High microbial gene richness; Stable microbiome functional cores	Related to gene, environmental and individual differences	30-34
Obesity	Decreased abundance: *Bacteroides thetaiotaomicron; Oscillospira; Methanobrevibacter smithii; Faecalibacterium prausnitzii* Elevated abundance: *Eubacterium ventriosum; Roseburia intestinalis*	Diet/ high BMI-related	38, 209-211
Type 2 diabetes (T2D)	Decreased abundance: *Clostridium spp*; *Akkermansia muciniphila; Bacteroides spp.*; *Butyrate-producing bacteria* Elevated abundance: *Dorea*; *Ruminococcus*; *Sutterella*; *Streptococcus; Akkermansia muciniphila*	Functional alterations in the metagenomes as causes or progression factor	39, 212-214
Atherosclerotic cardiovascular disease (ACVD)	Decreased abundance: *Prevotella* Elevated abundance: *Streptococcus*; *Escherichia*	Unknown	215
Cardio-metabolic diseases (CMD)	Decreased abundance: *Bacteroides spp*.; *Faecalibacterium prausnitzii*	Their metabolites blocking NF-κB activation and IL-8 secretion	216
Chronic heart failure (CHF)	Decreased abundance: *Alistipes*; *Faecalibacterium*; *Oscillibacter spp.* Elevated abundance: *Ruminococcus*; Acinetobacter; *Veillonella spp.*	Associated with altered fecal and plasma metabolic patterns	217
Non-alcoholic fatty liver disease (NAFLD)	Decreased abundance: *Oscillibacter*; *Flavonifaractor*; *Odoribacter*; *Alistipes spp.* Elevated abundance: *Clostridium*; *Anaerobacter*; Streptococcus; *Escherichia*; *Lactobacillus*;* Klebsiella pneumoniae*	Excess endogenous alcohol production; An elevated level of proinflammatory cytokines	40, 42
Non-alcoholic steatohepatitis (NASH)	Decreased abundance: *Oscillospira spp*. Elevated abundance: Proteobacteria; *Enterobacteriaceae*; *Escherichia spp.*; *Dorea*; *Ruminococcus spp.*	Excess endogenous alcohol production; Higher fecal concentrations of 2-butanone and 4-methyl-2-pentanone that cause hepatocellular toxicity	218, 219
Liver cirrhosis	Elevated abundance: Proteobacteria; Fusobacteria	An increase of microbial haem biosynthesis and phosphotransferase systems	220
Malnutrition, Severe acute malnutrition (SAM)	Decreased abundance: *Bifidobacterium longum*; *Bifidobacterium pseudolongum*	Related with not breastfeeding and unhealthy diet	221

**Table 2:** The effects of the gut microbiota/ its metabolites on ISCs in different intestinal states.

Gut microbiota/ metabolites	Model	Effects on ISCs	Mechanisms	References
Preterm infant gut microbiota	Humanized microbiome gnotobiotic mouse model	Promoting cell proliferation	Upregulating the expression of Cryptdin 5, Muc3 and Lyz1	29
Lactic acid producing bacteria	Normal mouse/ Drosophila	Promoting proliferation	Stimulating the Wnt/β-catenin pathway through GPR81; Nox-mediated generation of ROS	48, 89, 91, 92
	Gut damage in mice caused by radiation and methotrexate; Gut damage in drosophila (*Lactobacillus plantarum* overgrowth)	Promoting proliferation and enhancing regeneration	GPR81 activation and downstream regulation on Wnt3; Activating production of ROS by the intestinal Nox	48, 90
*Lactobacillus reuteri*	Normal mouse intestinal organoid	Promoting proliferation	Stimulating the Wnt/β-catenin pathway through increase in R-spondins	49
	TNF-induced intestinal organoid damage; C. rodentium-induced intestinal inflammation in mice	Promoting proliferation and differentiation	Activating the Wnt/β-catenin pathway and upregulating Wnt3 and Lrp5 expression	49
*Erwinia carotovora*	*E. carotovora* infection in Drosophila	Promoting proliferation and division	Initiating the JAK-STAT signaling	222
*Bacillus subtilis*	DSS-induced mouse colitis model	Protecting ISCs from inflammatory injury and inducing proliferation	Rebalancing the intestinal flora	52
SCFAs (acetate, butyrate, propionate)	Crypt culture	Butyrate: Promoting proliferation	Unknown	57
	Mice treated with vancomycin; Intestinal organoids	Valproic acid, Acetate, Propionate, and Butyrate: Promoting proliferation	Notch activating; MEK-ERK signaling; Inhibition of histone deacetylases by butyrate	58-61
	Primary colonic ISCs	Butyrate: Inhibitory effect Acetate, Propionate: No effect	Foxo3-dependent	62, 63
	Hydrochloric acid induced rat epithelial injury model	Butyrate: No effect	Unknown	64
Lactate (SCFAs intermediate metabolites)	Normal mouse; Mouse small intestine organoid; Gut damage caused by radiation or methotrexate	Promoting proliferation and regeneration	GPR81 stimulates of the Wnt/β-catenin signal pathway	48
Succinate (SCFAs intermediate metabolites)	Normal rat	Inhibiting proliferation	Unknown	69
Tryptophan metabolite	AhR^-/-^ mouse	Indoleacetic acid (IAA): Inhibiting proliferation	Suppresses β-catenin signals through AhR	77
	AhR^-/-^ mouse, Villin^Cre^Ahr^fl/fl^ mouse; Mouse intestinal organoid	Indole-3-carbinol (I3C): Inhibiting proliferation	Activating AhR	78, 80
	DSS-induced mouse IBD model	Indoleacrylic acid (IA): Promoting differentiation	Activates AhR to elevate expression of Ki67	81
	TNF-α-induced intestinal organoid injury; *Candida albicans* infected mouse model	Indolealdehyde (IAld): Promoting differentiation	Induction of IL-22	82, 83
Secondary bile acids: lithocholic acid (LCA), deoxycholic acid (DCA)	Human colon cancer cell model; Rat small intestinal crypt cells	Promoting proliferation at low dose, and inhibition at high dose	Promotion: Possible involvement of Wnt/β-catenin signaling Inhibition: EGFR and FXR signaling	86-88
Polyamines	Rat small intestinal crypt cells; Phytohaemagglutinin induced gut growth	Promoting proliferation	Inducing TGF-βRI; Increasing c-fos, c-myc, and c-jun expression; Acting as energy source	93-95

## Abbreviations

AhR: aryl hydrocarbon receptor
BMP: bone morphogenetic protein
CMD: cardio-metabolic disease
CF: cystic fibrosis
Cftr: cystic fibrosis transmembrane conductance regulator
CRC: colorectal cancer
DCA: deoxycholic acid
Dclk1: dual cortisol-like kinase 1
EC: epithelial cell
FGF: fibroblast growth factor
GI: gastrointestinal
GALT: gut-associated lymphoid tissue
GMMs: gut microbial metabolites
HFD: high-fat diet
ISC: intestinal stem cell
IBD: inflammatory bowel disease
IAA: indoleacetic acid
IA: indoleacrylic acid
Iald: indole aldehyde
I3C: indole-3-carbinol
LPS: lipopolysaccharides
LCA: lithocholic acid
NAFLD: non-alcoholic fatty liver disease
Nrf2: nuclear factor erythroid 2-related factor 2
NECD: Notch extracellular domain
NICD: Notch intracellular domain
PXR: pregnane X receptor
ROS: reactive oxygen species
SCFAs: short-chain fatty acids
SBAs: secondary bile acids
TJ: tight junction
TA cells: transit-amplifying cells
T2D: type 2 diabetes
TM: transmembrane
TEAD: transcriptional enhanced associate domain
TSC2: Tuberous sclerosis complex 2
TGF-β: transforming growth factor beta
2D: tow-dimensional
3D: three-dimensional
UC: ulcerative colitis
VDR: vitamin D receptor
